# Supplementary figures and images for: Inclusion of Oat Polar Lipids in a Solid Breakfast Improves Glucose Tolerance, Triglyceridemia, and Gut Hormone Responses Postprandially and after a Standardized Second Meal: A Randomized Crossover Study in Healthy Subjects
Source: Nutrients. 2023 Oct 16;15(20):4389. doi: 10.3390/nu15204389 (PMC10609583; doi:10.3390/nu15204389)

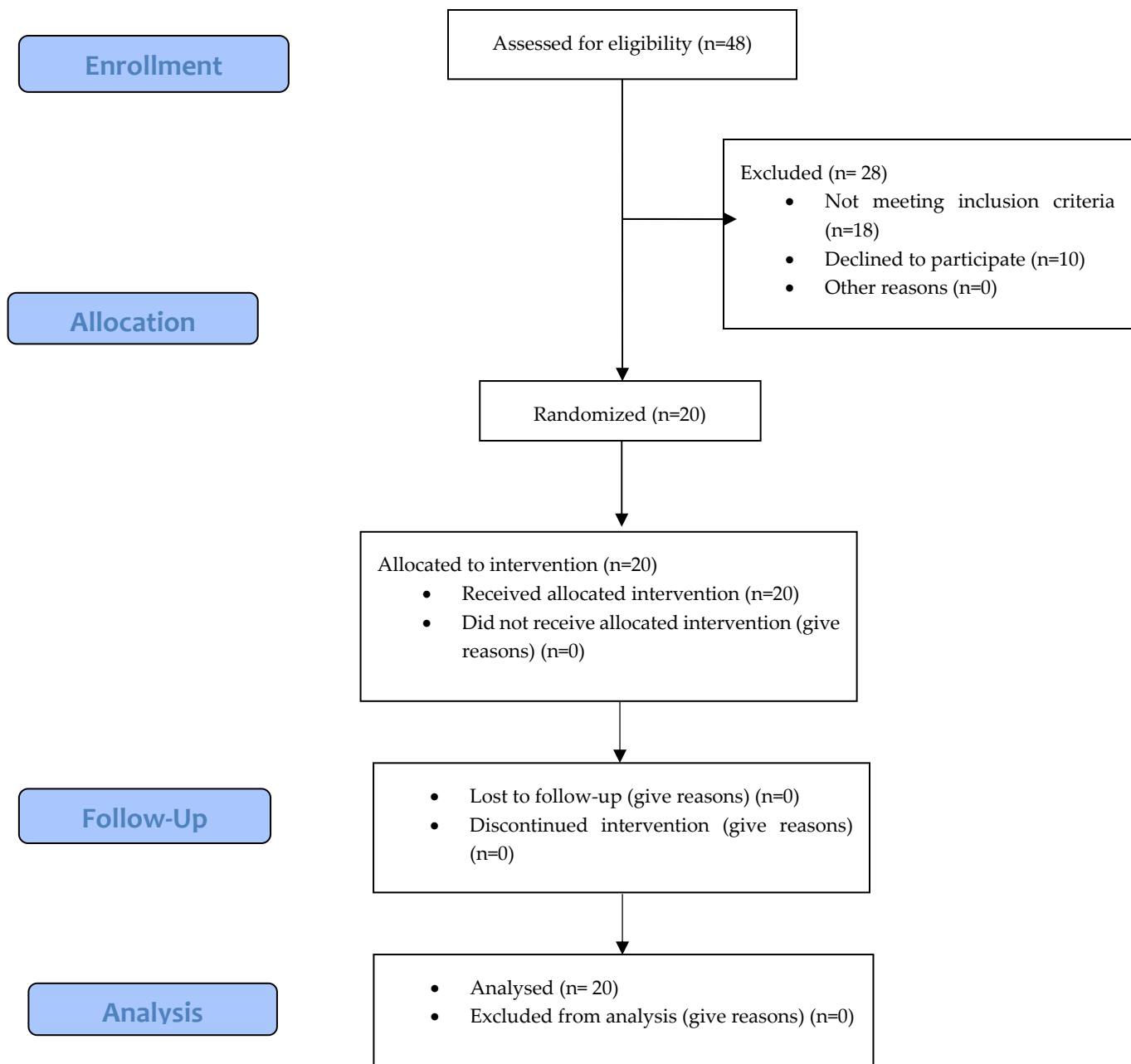

**Figure S1:** Flow diagram of the study progress

Supplement: Supplementary file 1 [file nutrients-15-04389-s001.zip › nutrients-2662084-supplementary.pdf]
